# Swallowing Outcomes in Open Partial Horizontal Laryngectomy Type I and Endoscopic Supraglottic Laryngectomy: A Comparative Study

**DOI:** 10.3390/ijerph19138050

**Published:** 2022-06-30

**Authors:** Carmelo Saraniti, Francesco Ciodaro, Cosimo Galletti, Salvatore Gallina, Barbara Verro

**Affiliations:** 1 Division of Otorhinolaryngology, Department of Biomedicine, Neuroscience and Advanced Diagnostic, University of Palermo, 90127 Palermo, Italy; carmelo.saraniti@unipa.it (C.S.); salvatore.gallina@unipa.it (S.G.); 2 Division of Otorhinolaryngology, Department of Adult and Development Age Human Pathology “Gaetano Barresi”, University of Messina, 98125 Messina, Italy; dottfciodaro@alice.it (F.C.); cosimogalletti92@gmail.com (C.G.)

**Keywords:** supraglottic laryngectomy, swallowing, partial laryngectomy, head and neck, surgical oncology

## Abstract

**Background:** Effective swallowing represents the main challenge in supraglottic laryngectomy. This study aimed to assess swallowing outcome comparing endoscopic supraglottic laryngectomy (ESL) and open partial horizontal laryngectomy type I (OPHL I). **Methods:** A retrospective study was carried out on 20 patients that underwent supraglottic laryngectomy from 2015 to 2021: 10 underwent ESL (group A) and 10 underwent OPHL I (Group B). Patients underwent fiberoptic endoscopic evaluation of swallowing (FEES) 3 months and 12 months after surgery and videofluoroscopy swallowing studies (VFSS) 12 months after surgery. A Swallowing Outcome After Laryngectomy (SOAL) questionnaire was administered to patients to assess their life quality. **Results:** A naso-gastric tube was placed in two patients of Group A and in all patients of Group B. Tracheostomy was performed in two patients of Group A and in all patients in Group B and it has been closed in 100% of them. According to Donzelli’s scale, FEES and VFSS showed better results in Group A at 3 months, while at 12 months they did not show statistically significant differences between ESL and OPHL I in terms of laryngeal penetration and aspiration. The SOAL questionnaire showed satisfactory life quality. **Conclusion:** Swallowing evaluation by FEES and VFSS did not demonstrate statistically significant differences at 12 months post-op between two surgeries, although ESL showed less cases of laryngeal penetration and aspiration at 3 months post-op. Anyway, good results of any surgery depend on careful patient selection and the surgeon’s experience.

## 1. Introduction

Squamous cell carcinoma (Scc) represents about 98% of laryngeal cancers. In particular, the supraglottis is the second most common laryngeal region affected by carcinoma (about 30%) [1].

In this laryngeal region, the tumor remains usually asymptomatic in the early stage; few and non-specific symptoms, as dysphagia or reflex otalgia, occur in advanced stage. Due to this attitude, diagnosis is often late and so prognosis of supraglottic cancer is poor [2]. Currently, T1-T2 supraglottic tumors can be treated by different strategies: radiotherapy (RT), transoral laser microsurgery (TLM) or open supraglottic laryngectomy (OPHL type I) [3,4]. The main goals of the treatment are: oncological radicality and organ and function preservation, in terms of swallowing, speaking and breathing. Recent studies found that TLM ensures organ preservation with poor impairment of laryngeal functions compared to other therapeutic approaches [3,5,6]. However, a careful pre-operative assessment of the patient is crucial for detection of the best therapeutic choice; in fact, it depends on the patient’s age, on his comorbidity and on tumor extension [7]. In particular, in 2009, the European Laryngological Society (ELS) proposed a classification of endoscopic supraglottic laryngectomy (ESL) [8] describing four types of TLM depending on tumor extension: limited excision of the small tumor (type I); medial supraglottic laryngectomy with partial resection of the pre-epiglottic space above (type IIa) or below (type IIb) hyoid bone; medial supraglottic laryngectomy with resection of the pre-epiglottic space for tumors extending to the petiole of epiglottis (type IIIa) or to ventricular folds (type IIIb); lateral supraglottic laryngectomy including the free edge of the epiglottis, threefolds’ region and ventricular folds (type Iva) or extending to the arytenoid (type IVb). This kind of surgery is indicated for T1-T2 tumors, without impairment of cordal function. On the other side, in 2014, ELS stated the classification of OPHL [9], defining three types: supraglottic (type I), supracricoid (type II) and supratracheal (type III) laryngectomy, depending on inferior limited resection. Moreover, in this case, the resection could extend to one arytenoid (ARY), base of tongue (BOT), one piriform sinus (PIR) or one crico-arytenoid unit (CAU).

The main challenge in supraglottic laryngectomy (ESL or open) is represented by valid and effective swallowing since the resected anatomical structures play an important role in airway protection during swallowing. So, in this study, we compared the two surgical techniques in order to define limits and advantages in terms of swallowing.

## 2. Materials and Methods

A retrospective study was carried out on patients that underwent supraglottic laryngectomy (ESL or open) in our ENT Clinics from January 2010 to December 2020. Informed consent to participate in the study was obtained from the patients and the study was approved by the ethics committee of our University Hospital. Inclusion criteria were: males and females, aged between 18 and 65 years old, with supraglottic Scc, T1-T2 according to criteria of the American Joint Committee on Cancer (AJCC), eligible for surgery (ESL type III–IV or OPHL type I), negative resection margin status and pN0. Concerning resection margin status, we assumed the cut-off of 1 mm as margin-tumor distance to distinguish between close and negative margins [10]. Exclusion criteria were: pre-operative and/or post-operative chemoradiation therapy (ChRT), previous other head and neck surgeries (except for the diagnostic biopsy), neurologic diseases, contraindication to fiberoptic endoscopic evaluation of swallowing (FEES) [11] and/or videofluoroscopy swallowing study (VFSS) [12] and previous oropharyngeal dysphagia. These strict criteria allowed to select patients of the two groups that were similar in all respects (age, sex, without pre-op and/or post-op ChRT and without other causes of impaired swallowing regardless of ELS or OPHL I). Doing so, we tried to avoid bias in the results due to the significant differences of the two groups that would have made the comparison of the two surgeries impossible.

### 2.1. Study Protocol

Recruited patients were divided into two groups depending on surgery: ESL (group A) and OPHL type I (group B). At a post-operative time, patients performed supraglottic swallowing exercises.

Patients underwent FEES twice: three months and one year after surgery. FEES was performed by the same dysphagia expert otorhinolaryngologist (CS). During the exam, we first tested laryngeal perception by the touch method [13]: we touch the laryngeal mucosa with the tip of the flexible laryngoscope in order to elicit a cough, gag or laryngeal adductor reflex and to evaluate any damage on the internal branch of superior laryngeal nerve. Recurrent laryngeal nerve function was assessed by phonatory exercises, breath holding and coughing. Then, the patient, in a sitting position, had to swallow two different food textures: liquid (water with green food dye) and thickened liquid (yogurt). The tip **of a flexible** endoscope was positioned beyond the soft palate and the pharyngeal phase of swallowing was studied recording videos and images for further analysis. In particular, according to the Donzelli secretion severity scale, the following parameters were assessed: pharyngeal residue, laryngeal penetration and tracheal aspiration. It is a three-point scale: level 1, functional, if there is pharyngeal residue without penetration or aspiration; level 2, severe, if secretion reaches laryngeal vestibule; level 3, profound, in case of tracheal aspiration [14,15].

One year after surgery, patients underwent VFSS: during the exam, patients had to swallow different textures of liquid (barium solution diluted with different volume of water) and antero-posterior and lateral fluoroscopic video images were recorded at a speed of 15 frames per second. These images were studied by the same radiologist with great experience in VFSS who investigated the same parameters—pharyngeal residue, laryngeal penetration, tracheal aspiration—according to Donzelli’s scoring scale.

One year after surgery, each patient was administered the Italian version of Swallowing Outcome After Laryngectomy (SOAL) questionnaire to assess their quality of life related to dysphagia [15,16]. This self-administered questionnaire consists of 17 items with multiple-choice answers: no; a little; a lot. Moreover, if patients answered “a little” or “a lot”, then they had to indicate if this bothered them.

Moreover, in the assessment of the functional outcome, duration of naso-gastric feeding, need of Percutaneous Endoscopic Gastrostomy (PEG) and/or persistence of tracheostomy had also been assessed.

### 2.2. Data Analysis

Demographic and medical data, type and subtype of surgery, tumor staging and grade of differentiation and resection margin status about recruited patients were collected in a data spreadsheet using the Microsoft Excel version 16.47.1. Results were reported as numbers, percentages of the total and/or mean ± standard deviation (SD). Chi-square tests with Yates correction were performed to compare the two groups (A and B) using MedCalc Statistical Software (MedCalc Software Ltd., Ostend, Belgium).

## 3. Results

The study included 20 patients: 4 females and 16 males, aged between 42 and 65 years old (mean age was 59.5 ± 4.91). Out of 20 patients, 10 underwent ESL (group A) and 10 underwent OPHL type I (Group B). In particular, Group A included: six type IIIa, two type IIIb and two type IVa. No endoscopic supraglottic laryngectomy type IVb was performed. Included patients had pT1-T2N0 supraglottic tumors with close or negative resection margins, so they did not need adjuvant therapy. Patients with close resection margins received a closer follow-up than patients with negative margins. Data are reported in Table 1.

### 3.1. FEES and VFSS

Three months after surgery, the touch method by endoscopy did not show impaired laryngeal perception in either group. Analyzing the FEES exam, Group A showed better results than Group B. In the former group, nobody had pharyngeal residue and/or tracheal aspiration. Only six patients (60%) had laryngeal penetration swallowing water (level 2 according to Donzelli’s score): two patients underwent ESL type IIIa, two patients ESL type IIIb and two patients ESL type IVa. As regards Group B, we found two patients with pharyngeal residue swallowing yogurt (level 1) and two patients with water in the laryngeal vestibule (level 2) and six patients with tracheal aspiration (level 3), according to Donzelli’s scale. At 12 months after surgery, eight patients (80%) of Group A showed a worsening swallowing, while on the contrary four patients (40%) of Group B had an improvement in swallowing and the other six (60%) showed no changes. VFSS, performed twelve months after surgery, showed the same results of FEES as regards Group A; instead, in Group B, we found the following results: level 1 in two patients, level 2 in two patients and level 3 in six patients. So, for Group B, FEES and VFSS findings were not perfectly overlapping, with more episodes of tracheal aspiration during VFSS. FEES and VFSS results are reported in Table 2.

Comparing the *food in laryngeal vestibule* parameter between the two groups based on the FEES results, we did not find statistically significant relationships between this parameter and the type of surgery (ESL vs. OPHL I): the chi-square statistic with Yates correction was 0.952 (*p*-value 0.32) and 0.312 (*p*-value 0.57), respectively, 3 months and 12 months after surgery.

### 3.2. Post-Operative Outcome

A naso-gastric tube was placed only in two patients of Group A (#2 and #5) and in all patients of Group B and removed on the 14th post-operative day in Group B, on the 4th post-operative day in patient #2 and on the 2nd post-operative day in patient #5. No patients needed a PEG. A tracheostomy was performed in all patients in Group B and it has been closed in 100% of them. As for Group A, a covering tracheostomy was performed in two patients (#2 and #5) during ESL: one patient underwent ESL type IIIa and one patient underwent ESL type IVa. In these two cases, covering tracheostomy was performed due to a swelling airway after surgery. However, the tracheostomy was closed a few weeks after surgery. It should be noted that, during the first FEES, swallowing worsened in Group A and was found in these two tracheostomized patients.

Hospitalization lasted less in case of ESL (12.5 ± 6.15). Post-operative bleeding occurred in only two patients, both of Group B. No other complications have been detected.

### 3.3. SOAL Questionnaire

Overall, the SOAL questionnaire showed a good and satisfactory quality of life without an impairment due to swallowing problems (Table 3). In particular, patients (6/20) reported problems swallowing thin liquids (item #2) and dry solid food (item #5). Of these six patients, four belonged to the Group B. Moreover, few patients answered that they took longer to eat a meal (item #12) and reduced the size of their meal (item #14). In conclusion, these swallowing problems did not affect their quality of life.

## 4. Discussion

There are several treatments for T1-T2 supraglottic cancer: radiotherapy, TLM, transoral robotic surgery (TORS) and open partial horizontal laryngectomy (OPHL). Each therapeutic approach has proven effective in terms of survival rates and local control: in particular, five-year local control rates range from about 75% to 100% and from about 60% to 80%, respectively, for T1 and for T2 supraglottic cancers regardless of the therapy [17]. As recommended in 2018 by the American Society of Clinical Oncology (ASCO), the best treatment for limited-stage (T1-T2) laryngeal cancer should have three goals: curing the cancer, preserving organ functions, and ensuring satisfactory quality of life [18]. The ASCO also stated that this optimal treatment can be achieved through a full and multidisciplinary evaluation of patients in terms of cancer staging and tumor characteristics (tumor size, local-regional extension), health and socioeconomic, psychosocial state and choice of patient and logistic issue [8,18,19,20]. Moreover, the treatment choice depends on surgeon expertise and availability of rehabilitative experts. However, the main and final issue is to avoid combined-modality therapy (surgery plus RT) because it can impair functional outcomes. According to these international guidelines and recommendations, we performed ELS or OPHL type I, keeping in mind that, also in experienced hands, the therapeutic choice depends mainly on laryngeal exposure to achieve tumor-free margins [8,18]. The open horizontal supraglottic laryngectomy was first described by Alonso in 1947 [21]. About 30 years later, in 1972, Strong et al. described the endoscopic supraglottic laryngectomy for the first time [22]. Moreover, a key problem related to both surgical and non-surgical treatments is impairment of swallowing. So, it represents the main challenge of surgical treatment for supraglottic tumors. For this reason, we decided to assess swallowing impairment and improvement in two group of patients: Group A underwent ESL and Group B underwent OPHL type I. We analyzed their swallowing residual function by FEES and VFSS exams, performed 3 months and 12 months after surgery. We found satisfying results in both groups, better in ESL patients. Moreover, we found no statistically significant different results at 12 months and an improvement in Group B. So, our study demonstrates that swallowing recovery is slower but similar in the case of OPHL than ESL. This finding is consistent with literature [7] and it could be explained by the reduced invasiveness of TLM, without sutures, strap muscles and thyroid cartilage involved and neither routine tracheostomy. Indeed, in our sample, ESL patients, who needed a covering tracheostomy, had worse swallowing at the beginning, probably due to the tracheostomy itself. The good results in both groups could also be explained by the preservation of the superior laryngeal nerve achieved in all patients.

Contrary to Peretti et al.’s study [23], we did not find any statistically significant differences between two therapeutic approaches for FEES. Further, in our study, tracheal aspiration was more frequent in VFSS than in FEES. Actually, although VFSS does not provide anatomic and function details, it can detect minor aspiration events. For this reason, the two exams are necessary and complementary for a comprehensive assessment of swallowing. In this regard, a study reported that, in patients undergoing supraglottic laryngectomy, tracheal aspiration events were more frequent post-swallowing rather than intra- or pre-swallowing [24].

In our study, in addition to the objective analysis of swallowing (FEES and VFSS), we also performed a subjective evaluation of the quality of life related to swallowing. The one-year SOAL questionnaire—created specifically for laryngectomized patients—showed good and satisfactory quality of life in both groups, although with minor swallowing impairment that did not affect their lives anyway. Similar results found Peretti et al. administering the M.D. Anderson Dysphagia Inventory (MDADI) questionnaire [23,25]. However, we should point out that results of the questionnaire between both groups cannot be compared because they are two different surgical techniques and patients’ lives facing two different swallowing difficulties and problems.

In our study, we evaluated the duration of naso-gastric feeding. In several studies, the naso-gastric tube was placed in almost all ESL patients [7,24,26]. On the contrary, we placed a naso-gastric tube only in two ESL patients and it was removed early. Additionally, a tracheostomy was performed in just two ESL patients due to swelling airways. However, we closed the tracheostomy on both of the patients. No patient, in either group, needed a permanent tracheostomy. This result is quite consistent with current literature [7,23]. In our case series, hospitalization lasted on average 12.5 ± 6.15 days and 27.5 ± 5.83 days for Group A and B, respectively: as expected and reported in literature [7,23], ESL has a faster recovery with a lower mean hospitalization than OPHL. Moreover, like all surgeries, supraglottic laryngectomies also run the risk of complications: post-surgical bleeding, neck abscess, chondritis of thyroid cartilage, aspiration pneumonia, laryngeal stenosis and pharyngocutaneous fistulas [7]. Aspiration pneumonia, need of tracheostomy and bleeding represent the main complications reported in literature. In our case series, we encountered only two cases of post-operative bleeding, both in Group B, and as reported above, two cases of temporary tracheostomy due to post-operative laryngeal swelling in Group A. No other complications have been encountered. Current literature did not demonstrate any statistically significant difference in aspiration pneumonia rate between these two types of surgeries [7,26].

Anyway, our study had a main limit: the small sample size. So, our results need to be confirmed by other studies. Indeed, to achieve the most homogeneous sample possible we included only patients who underwent ESL type III or IV with a wider resection, comparable to OPHL. Moreover, we have excluded patients over the age of 65 in order to avoid bias related to presbyphagia. For the same reason, we also excluded patients who underwent pre-operative and/or post-operative ChRT because it can impair swallowing. FEES and VFSS were always performed by the same specialists in order to avoid bias related to different performance of the examination and interpretation of the results.

## 5. Conclusions

Supraglottic carcinoma surgery, both endoscopic and transcervical, was born with the main aim of reaching oncological radicality with safe resection margins and, at the same time, the preservation of laryngeal functions. From our analysis of the swallowing outcome, endoscopic surgery resulted better at 3 months post-op, but 12 months swallowing results did not show statistically significant differences in terms of laryngeal penetration and aspiration between the two surgeries. Anyway, good and appreciated oncological and functional results of each surgical technique depend on two main factors: careful patient selection and the surgeon’s experience.

## 6. Highlights

- The main goals of supraglottic carcinoma surgery are oncological radicality and organ and function preservation;
- Supraglottic carcinoma surgery can be endoscopic (ESL) or transcervical (OPHL I);
- Swallowing results are better in ESL at 3 months post-op, but at 12 months post-op the two surgeries do not show statistically significant different results;
- Results of surgical techniques depend on careful patient selection and the surgeon’s experience.

## Figures and Tables

**Table 1:** Patients’ characteristics.

Characteristics		N (%)
*Sex*		
Male		16 (80)
Group A		10
Group B		6
Female		4 (20)
Group A		0
Group B		4
*Age (years)*		
Mean (± SD)		59.5 ± 4.91
Group A		60.8 ± 3.25
Group B		58.3 ± 5.88
Range		42–65
Group A		56–65
Group B		60–65
*ESL (Group A)*		
Type IIIa		6 (60)
Type IIIb		2 (20)
Type IVa		2 (20)
Type IVb		0 (0)
*OPHL I (Group B)*		10
*Superior laryngeal nerve integrity (touch method)*		
Group A		10
Group B		10
*Duration of naso-gastric feeding (days)*		
Group A		
Patient #2		2
Patients #5		4
Group B		14
*PEG*		
Group A		0
Group B		0
*Need of tracheostomy*		
Group A		2
Group B		10
*Permanent tracheostomy*		
Group A		0
Group B		0
Hospitalization time (days)		
Mean ± SD		
Group A		12.5 ± 6.15
Group B		27.5 ± 5.83
Range		
Group A		6–26
Group B		18–45
*Post-op complications*	Group A	Group B
Bleeding	0	2 (20)
Prelaryngeal abscess	0	1 (10)
Edema	2 (20)	0
Chondritis	0	0
Aspiration pneumonia	0	0
Laryngeal stenosis	0	0
*pT*	Group A	Group B
T1	7	5
T2	3	5
Grade of differentiation	Group A	Group B
G1	3	0
G2	4	6
G3	3	4
*Resection margin status*	Group A	Group B
Close (<1 mm)	4	3
Negative (>1 mm)	6	7
Total		20 (100)

SD: standard deviation; ESL: endoscopic supraglottic laryngectomy; OPHL: open partial horizontal laryngectomy; PEG: Percutaneous Endoscopic Gastrostomy; pT: pathological stage of tumor; G1: low grade or well-differentiated tumor; G2: moderate grade or moderately differentiated tumor; G3: high grade or poorly differentiated tumor.

**Table 2:** FEES and VFSS results.

Group	Sex	Age	Surgery	FEES		VFSS
Group A			ELS	3 Months *	12 Months *	12 Months *
#1	M	58	IIIa	No dysphagia	Level 1	Level 1
#2	M	56	IVa	Level 2	Level 2	Level 2
#3	M	65	IIIb	Level 2	Level 3	Level 3
#4	M	63	IIIa	No dysphagia	Level 2	Level 2
#5	M	65	IIIa	Level 2	Level 3	Level 3
#6	M	58	IIIb	Level 2	Level 3	Level 3
#7	M	57	IIIa	No dysphagia	Level 1	Level 1
#8	M	64	IIIa	No dysphagia	Level 2	Level 2
#9	M	60	IVa	Level 2	Level 2	Level 2
#10	M	62	IIIa	Level 2	Level 3	Level 3
**Group B**						
#11	M	60	OPHL I	Level 3	Level 2	Level 3
#12	F	42	OPHL I	Level 2	Level 2	Level 2
#13	F	60	OPHL I	Level 1	Level 1	Level 1
#14	M	62	OPHL I	Level 3	Level 2	Level 3
#15	M	65	OPHL I	Level 3	Level 3	Level 3
#16	M	56	OPHL I	Level 2	Level 2	Level 2
#17	F	60	OPHL I	Level 1	Level 1	Level 1
#18	M	59	OPHL I	Level 3	Level 2	Level 3
#19	F	58	OPHL I	Level 3	Level 2	Level 3
#20	M	61	OPHL I	Level 3	Level 3	Level 3

* after surgery.

**Table 3:** Swallowing outcome after laryngectomy (SOAL) questionnaire [16] (one year after surgery).

	Items	Not	A Little	A Lot	If You Answered “a Little” or “a Lot”, Please Indicate if This Bothers You
1	*In your opinion, do you have a swallowing problem now?*	16	4	0	0
2	*Do you have a problem swallowing thin liquids (water…)?*	14	6	0	0
3	*Do you have a problem swallowing thick liquids (soup…)?*	20	0	0	-
4	*Do you have a problem swallowing soft/mashed foods?*	20	0	0	-
5	*Do you have a problem swallowing dry solid food (bread…)?*	14	6	0	0
6	*Do liquids stick in your throat when you swallow?*	18	2	0	0
7	*Does food stick in your throat when you swallow?*	16	4	0	0
8	*Does food or liquid come back up into your mouth or nose when you eat or drink?*	20	0	0	-
9	*Do you need to swallow liquid to help the food go down?*	18	2	0	0
10	*Do you need to swallow many times on each mouthful to help the food or drink go down?*	18	20	0	0
11	*Do you avoid certain food because you cannot swallow them?*	16	4	0	0
12	*Does it take longer to eat a meal?*	14	6	0	0
13	*Has your enjoyment of food reduced?*	20	0	0	-
14	*Has the size of your meal reduced?*	12	8	0	0
15	*Has your appetite reduced because you cannot taste or smell food normally?*	20	0	0	-
16	*Has your eating been more difficult due to dry mouth?*	16	4	0	0
17	*Do you feel self-conscious eating with other people?*	18	2	0	0

Value reported as numbers.

## Data Availability

Not applicable.

## References

[1] Nocini R., Molteni G., Mattiuzzi C., Lippi G. (2020). Updates on larynx cancer epidemiology. Chin. J. Cancer Res..

[2] Patel T.D., Echanique K.A., Yip C., Hsueh W.D., Baredes S., Park R.C.W., Eloy J.A. (2018). Supraglottic Squamous Cell Carcinoma: A Population-Based Study of 22,675 Cases. Laryngoscope.

[3] Ambrosch P., Gonzalez-Donate M., Fazel A., Schmalz C., Hedderich J. (2018). Transoral Laser Microsurgery for Supraglottic Cancer. Front. Oncol..

[4] Verro B., Greco G., Chianetta E., Saraniti C. (2020). Management of Early Glottic Cancer Treated by CO_2_ Laser According to Surgical-Margin Status: A Systematic Review of the Literature. Int. Arch. Otorhinolaryngol..

[5] Swanson M., Low G., Sinha U.K., Kokot N. (2017). Transoral surgery vs intensity-modulated radiotherapy for early supraglottic cancer: A systematic review. Curr. Opin. Otolaryngol. Head Neck Surg..

[6] Saraniti C., Montana F., Chianetta E., Greco G., Verro B. (2020). Impact of resection margin status and revision transoral laser microsurgery in early glottic cancer: Analysis of organ preservation and local disease control on a cohort of 153 patients. Braz. J. Otorhinolaryngol..

[7] Estomba C.C., Reinoso F.B., Lorenzo A.L., Conde J.F., Nores J.A., Hidalgo C.S. (2016). Functional outcomes of supraglottic squamous cell carcinoma treated by transoral laser microsurgery compared with horizontal supraglottic laryngectomy in patients younger and older than 65 years. Risultati funzionali in pazienti over e under 65 affetti da carcinoma squamocellulare sopraglottico trattati con chirurgia laser trans-orale o tradizionale laringectomia orizzontale sopraglottica. Acta Otorhinolaryngol. Ital..

[8] Remacle M., Hantzakos A., Eckel H., Evrard A.-S., Bradley P.J., Chevalier D., Djukic V., de Vincentiis M., Friedrich G., Olofsson J. (2009). Endoscopic supraglottic laryngectomy: A proposal for a classification by the working committee on nomenclature, European Laryngological Society. Eur. Arch. Otorhinolaryngol..

[9] Succo G., Peretti G., Piazza C., Remacle M., Eckel H.E., Chevalier D., Simo R., Hantzakos A.G., Rizzotto G., Lucioni M. (2014). Open partial horizontal laryngectomies: A proposal for classification by the working committee on nomenclature of the European Laryngological Society. Eur. Arch. Otorhinolaryngol..

[10] Osuch-Wójcikiewicz E., Rzepakowska A., Sobol M., Bruzgielewicz A., Niemczyk K. (2019). Oncological outcomes of CO_2_ laser cordectomies for glottic squamous cell carcinoma with respect to anterior commissure involvement and margin status. Lasers Surg. Med..

[11] Langmore S.E. (2017). History of Fiberoptic Endoscopic Evaluation of Swallowing for Evaluation and Management of Pharyngeal Dysphagia: Changes over the Years. Dysphagia.

[12] Kim S.Y., Kim T.U., Hyun J.K., Lee S.J. (2014). Differences in Videofluoroscopic Swallowing Study (VFSS) Findings according to the Vascular Territory Involved in Stroke. Dysphagia.

[13] Kaneoka A., Pisegna J., Inokuchi H., Ueha R., Goto T., Nito T., Stepp C.E., LaValley M., Haga N., Langmore S.E. (2017). Relationship Between Laryngeal Sensory Deficits, Aspiration, and Pneumonia in Patients with Dysphagia. Dysphagia.

[14] Donzelli J., Wesling M., Brady S., Craney M. (2003). Predictive Value of Accumulated Oropharyngeal Secretions for Aspiration during Video Nasal Endoscopic Evaluation of the Swallow. Ann. Otol. Rhinol. Laryngol..

[15] Saraniti C., Speciale R., Santangelo M., Massaro N., Maniaci A., Gallina S., Serra A., Cocuzza S. (2019). Functional outcomes after supracricoid modified partial laryngectomy. J. Biol. Regul. Homeost. Agents.

[16] Govender R., Lee M., Davies T., Twinn C., Katsoulis K., Payten C.L., Stephens R., Drinnan M. (2012). Development and preliminary validation of a patient-reported outcome measure for swallowing after total laryngectomy (SOAL questionnaire). Clin. Otolaryngol..

[17] Jones T.M., De M., Foran B., Harrington K., Mortimore S. (2016). Laryngeal cancer: United Kingdom National Multidisciplinary guidelines. J. Laryngol. Otol..

[18] Forastiere A.A., Ismaila N., Lewin J., Nathan C.A., Adelstein D.J., Eisbruch A., Fass G., Fisher S.G., Laurie S.A., Le Q.-T. (2018). Use of Larynx-Preservation Strategies in the Treatment of Laryngeal Cancer: American Society of Clinical Oncology Clinical Practice Guideline Update. J. Clin. Oncol..

[19] Saraniti C., Verro B., Ciodaro F., Galletti F. (2022). Oncological Outcomes of Primary vs. Salvage OPHL Type II: A Systematic Review. Int. J. Environ. Res. Public Health.

[20] Sperry S.M., Rassekh C.H., Laccourreye O., Weinstein G.S. (2013). Supracricoid Partial Laryngectomy for Primary and Recurrent Laryngeal Cancer. JAMA Otolaryngol. Neck Surg..

[21] Alonso J.M. (1947). Conservative surgery of cancer of the larynx. Trans. Am. Acad. Ophthalmol. Otolaryngol..

[22] Strong M.S., Jako G.J. (1972). Laser Surgery in the Larynx Early Clinical Experience with Continuous CO_2_ Laser. Ann. Otol. Rhinol. Laryngol..

[23] Peretti G., Piazza C., Cattaneo A., De Benedetto L., Martin E., Nicolai P. (2006). Comparison of Functional Outcomes after Endoscopic versus Open-Neck Supraglottic Laryngectomies. Ann. Otol. Rhinol. Laryngol..

[24] Prgomet D., Bumber Z., Bilić M., Svoren E., Katić V., Poje G. (2002). Videofluoroscopy of the swallowing act after partial supraglottic laryngectomy by CO_2_ laser. Eur. Arch. Otorhinolaryngol..

[25] Chen A.Y., Frankowski R., Bishop-Leone J., Hebert T., Leyk S., Lewin J., Goepfert H. (2001). The development and validation of a dyspha-gia-specific quality-of-life questionnaire for patients with head and neck cancer: The M. D. Anderson dysphagia inventory. Arch. Otolaryngol. Head Neck Surg..

[26] Cabanillas R., Rodrigo J.P., Llorente J.L., Suárez V., Ortega P., Suárez C. (2004). Functional outcomes of transoral laser surgery of supraglottic carcinoma compared with a transcervical approach. Head Neck.

